# Translating cell mechanobiology and nuclear deformations to the clinic

**DOI:** 10.1002/ctm2.1000

**Published:** 2022-07-24

**Authors:** Yohalie Kalukula, Marine Luciano, Sylvain Gabriele

**Affiliations:** ^1^ Mechanobiology & Biomaterials group, Interfaces and Complex Fluids Laboratory, Research Institute for Biosciences, CIRMAP University of Mons Mons Belgium; ^2^ Department of Biochemistry University of Geneva Geneva Switzerland

1

For more than a century, pathologists have used the nucleus as a diagnostic tool based on the observation of morphologic abnormalities.^1^ More recently, emerging evidence has pointed to a new route to determine the metastatic potential of cancer cells by focusing on changes in cell stiffness as a new biomarker. These features are key to establishing a diagnosis of malignancy in cytopathology and surgical pathology. Cytopathologists have developed a unique ability to distinguish between benign and malignant cells in tissue biopsies by detecting with the eye cells increased nuclear size, irregularities of the nuclear membrane, or modifications of chromatin distribution. Despite the extensive use of morphological abnormalities of the nucleus as a powerful diagnostic tool, the underlying mechanisms remain unclear and associated changes in the mechanical properties of cells are still poorly examined. Moreover, automated image analysis of nuclear shape and state‐of‐the‐art microfabricated tools to probe cell and nuclear deformability has not been translated to the clinics yet.

Recent evidence has highlighted the key role of the nuclear envelope in regulating nuclear mechanics and mechanotransduction,^2^ which refers to the molecular process in which mechanical stimuli are converted into biochemical signals and is more broadly used to define cellular responses to changes in the mechanical environment in terms of forces, deformations or mechanical properties. Indeed, defects in the nuclear lamina protein network that underlies the inner nuclear membrane can result in impaired nuclear stability, increased nuclear fragility and perturbations of mechanotransduction pathways. These defects are associated with Lamin A mutations linked to dilated cardiomyopathy, muscular dystrophy, some types of cancer, Hutchinson‐Gilford progeria syndrome as well as increased DNA damage. In addition, recent works have shown that lamins have a key role in tethering and organizing chromatin and in signalling involved in transcriptional regulation.

The regulation of the nuclear morphology is related to a physical coupling between the cytoskeleton and the nuclear lamina, which is achieved by the linker of nucleoskeleton and cytoskeleton (LINC) complexes. Additional mechanisms, such as molecular motors binding to nuclear pore complexes or microtubules connecting to emerin and other nuclear envelope proteins, may further contribute to nucleo‐cytoskeletal coupling. Due to this physical coupling, cytoskeletal forces exerted on the nuclear lamina can severely deform the nuclear envelope and trigger the reversible formation of heterochromatin, epigenetic changes (H3K9me3 methylation) or chromatin unpacking.^3^, ^4^ A large amount of contractile tension in actin stress fibres can lead to nuclear indentations of a few microns, which are characterized by the local enrichment of LINC complexes and can lead to segregated domains of condensed chromatin.^5^ In addition to promoting large nuclear deformations, loss of lamins can result in a local mechanical fragility that leads to an increased propensity for nuclear envelope rupture, which describes the transient loss of nuclear membrane integrity at a localised site.^6^ Nuclear envelope rupture events are transient events that can cause DNA damage by allowing access of the ER associated exonuclease TREX1 into the nucleus or by loss of DNA damage repair factors from the nucleus via nuclear efflux.

Nuclear deformability and changes in mechanical properties of cells are a hallmark of important physiological and pathological situations, such as the migration of immune cells that must navigate through narrow interstitial spaces of few microns or tumoral cancer cells that migrate in confined microenvironments when invading tissues and intra‐ or extravasating blood vessels to metastasize to distant tissues.^2^ The ability to interrogate nuclear and cellular mechanical properties as biophysical markers of disease, which in some instances may present earlier and be more specific than biological signs of disease, can drastically enhance the accuracy of disease diagnoses and inform about disease management from a patient‐centric perspective.

To this aim, lab‐on‐a‐chip microsystems based on protein micropatterns,^7^ microstructured soft hydrogels ^8^ or microfluidic channels ^9^ are poised to substantially impact the biomedical field by providing low‐cost, standardized and high throughput devices that can analyze disease‐mediated biophysical cellular changes in the clinical setting in order to diagnose patients and monitor disease prognosis (Figure 1A). The possibility to tailor lab‐on‐a‐chip devices for a specific experimental design enhances their ability to interrogate biophysical markers of disease such as nuclear deformability or modulation of the cellular mechanics (Figure 1B) and elucidate sample heterogeneity. Remarkably, lab‐on‐a‐chip microsystems can be easily combined with complementary sequencing techniques such as ChIP (chromatin immunoprecipitation) to provide information about pathology‐related changes in transcriptional programs (Figure 1C). In addition, automated image analysis programs can enable efficient real‐time or post‐experiment analyses of morphological and mechanical features in a high throughput manner, allowing processing to be complete within a few hours (Figure 1D). Beyond the translation of lab‐on‐a‐chip microsystems to the clinics, being able to automate cellular and nuclear detection could serve as a useful tool in clinical practice to make better decisions in diagnosis, prognosis, and treatment by lowering the time‐consuming aspect of laborious manual analyses, which can be highly subjective (Figure 1E). In this way, artificial intelligence techniques and machine learning solutions are promising candidates to streamline the process of cell and nuclear detection in tissue biopsies (Figure 1F,G) that contain rich information ‐ but usually under‐exploited ‐ and where nuclei appear crowded.^10^

**FIGURE 1:**
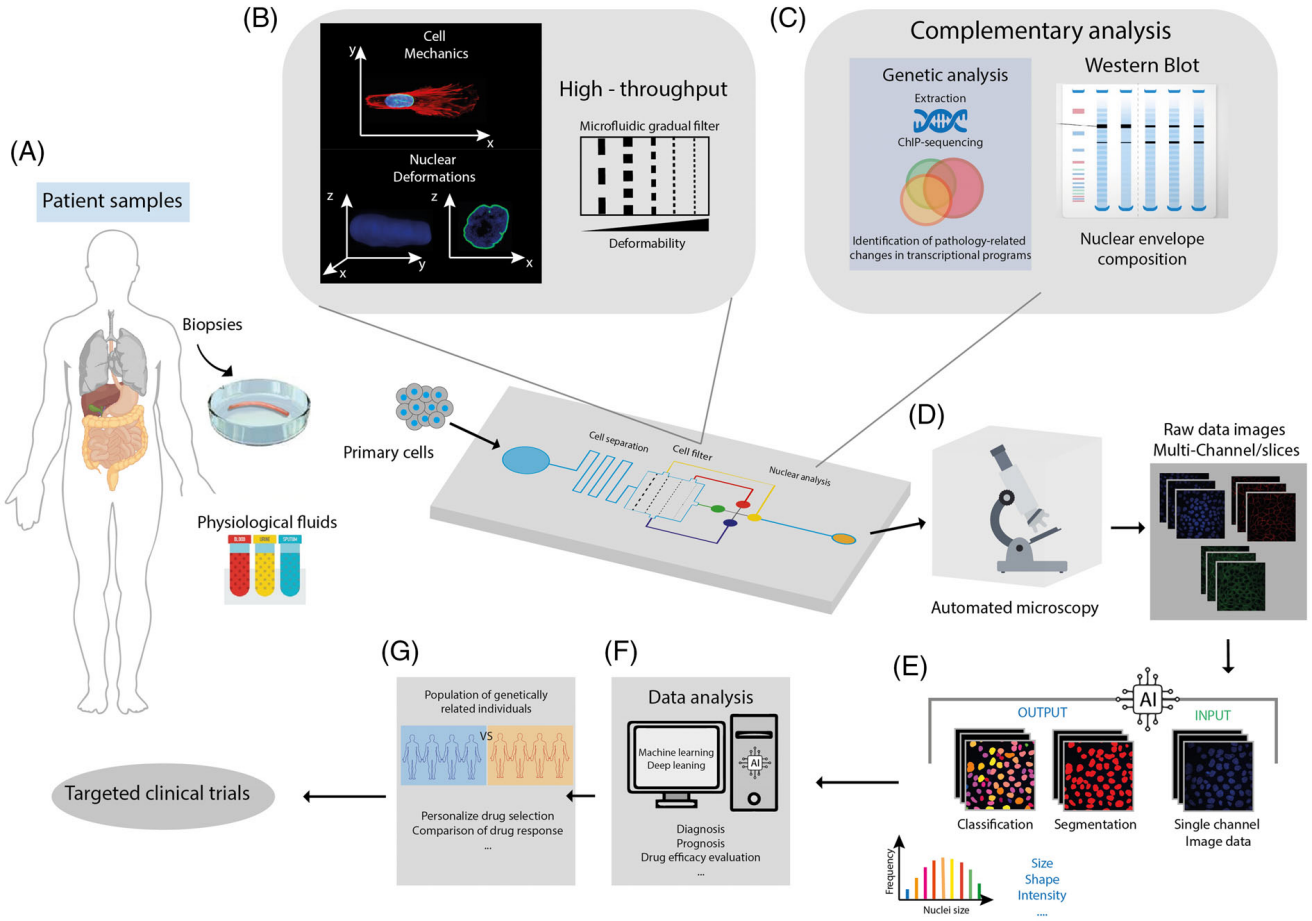
Lab‐on‐a‐chip device designed for clinical applications and using mechanical and nuclear deformability as biomarkers. (A) Primary cells are obtained from patient biopsies or physiological fluids (blood, lymph, urine). Large amounts of isolated cells are injected into the chip to perform high throughput analysis. (B) The mechanical deformability of cells and their nuclei can be used as biomarkers. First, cells pass through a cell separation channel to isolate the nucleus from the cytoplasm. Then, nuclei can be sorted according to their deformability level using a microfluidic gradual filter. In each compartment, nuclei fractions can be harvested for further analysis. (C) Complementary analyses, such as western blots, can be conducted to obtain nuclear envelope composition (lamins levels). Sequencing techniques, such as ChIP (chromatin immunoprecipitation), can provide information about pathology‐related changes in transcriptional programs. (D) Automated microscopy acquisition was conducted on sorted nuclei using confocal microscopy. (E) Artificial intelligence (AI) can be used to treat raw image data and classify nuclei according to their size, shape, and fluorescence intensity of a specific marker. (F) Machine learning and deep learning can be used to infer prognosis, and potential diagnosis and even identify drug treatment efficacy. (G) Lab‐on‐a‐chip devices can be tailored to study specific patient profiles and design personalize drug treatments or clinical trials

The field of cell mechanobiology and the focus on nuclear mechanics is emerging into a period that has rich potential for clinical translation. The ability to interrogate biophysical markers of disease, which in some instances may present earlier and be more specific than biological signs of disease, can drastically enhance the accuracy of disease diagnoses and inform disease management from a patient‐centric perspective. The possibility to implement alone or in conjunction with gold‐standard assays like flow cytometry gives lab‐on‐a‐chip microsystems substantial promise to be transformative technologies in the clinic. There are many encouragements for the translation of innovative works from diverse sources ranging from academic institutions, government agencies and private investors. Academics have therefore the opportunity to contribute to translational research in multiple ways and the possibilities for cell mechanobiology to impact society through a translation to the clinics are impressive.

## CONFLICT OF INTEREST

The authors declare no conflict of interest.

## References

[1] de Lad Heras JI , Schirmer EC . The nuclear envelope and cancer: a diagnostic perspective and historical overview. Adv Exp Med Biol. 2014;773:5‐26. doi:10.1007/978-1-4899-8032-8_1 24563341

[2] Kalukula Y , Stephens AD , Lammerding J , Gabriele S . Mechanics and functional consequences of nuclear deformations. Nat Rev Mol Cell Biol. Published online May 5, 2022. doi:10.1038/s41580-022-00480-z PMC9902167 35513718

[3] Versaevel M , Grevesse T , Gabriele S . Spatial coordination between cell and nuclear shape within micropatterned endothelial cells. Nat Commun. 2012;3:671. doi:10.1038/ncomms1668 22334074

[4] Tajik A , Zhang Y , Wei F , et al. Transcription upregulation via force‐induced direct stretching of chromatin. Nat Mater. 2016;15:1287‐1296. doi:10.1038/nmat4729 27548707 PMC5121013

[5] Versaevel M , Braquenier J‐B , Riaz M , et al. Super‐resolution microscopy reveals LINC complex recruitment at nuclear indentation sites. Sci Rep. 2015;4:7362. doi:10.1038/srep07362 PMC4258653 25482017

[6] Denais CM , Gilbert RM , Isermann P , et al. Nuclear envelope rupture and repair during cancer cell migration. Science. 2016;352:353‐358. doi:10.1126/science.aad7297 27013428 PMC4833568

[7] Mohammed D , Charras G , Vercruysse E , et al. Substrate area confinement is a key determinant of cell velocity in collective migration. Nat Phys. 2019;15:858‐866. doi:10.1038/s41567-019-0543-3

[8] Luciano M , Xue S‐L , De Vos WH , et al. Cell monolayers sense curvature by exploiting active mechanics and nuclear mechanoadaptation. Nat Phys. 2021;17:1382‐1390. doi:10.1038/s41567-021-01374-1

[9] Preira P , Grandné V , Forel J‐M , et al. Passive circulating cell sorting by deformability using a microfluidic gradual filter. Lab Chip. 2013;13:161‐170. doi:10.1039/C2LC40847C 23147069

[10] Phillip JM , Han K‐S , Chen W‐C , et al. A robust unsupervised machine‐learning method to quantify the morphological heterogeneity of cells and nuclei. Nat Protoc. 2021;16:754‐774. doi:10.1038/s41596-020-00432-x 33424024 PMC8167883

